# Predictors of dual protection use based on information-motivation-behavior skill model among female university students: a cross-sectional study

**DOI:** 10.3389/frph.2025.1407854

**Published:** 2025-08-14

**Authors:** Banti Negero Feyisa, Gurmesa Tura Debelew, Zewdie Birhanu Koricha

**Affiliations:** ^1^ Department of Population and Family Health, Jimma University, Jimma, Ethiopia; ^2^ Department of Health Behavior and Society, Jimma University, Jimma, Ethiopia

**Keywords:** IMB model, HIV risk reduction, safer sexual dual protection behavior, consistent condom use, dual protection use, female university students

## Abstract

**Background:**

The burden of sexually transmitted infections and unintended pregnancy remain a major health problem disproportionately affecting young woman in sub Saharan Africa. While there is a growing interest in promoting dual protection as a means of simultaneously preventing both HIV/STIs and unwanted pregnancy, little is known about patterns and predictors of dual protection use based on theoretical models for designing targeted interventions to promote dual protection for youth. This study aimed to examine predictors of dual protection use based on the information-motivation-behavioral skills model among female university students in Ethiopia.

**Methods:**

A cross-sectional study was conducted with 1,020 female students at Mattu University between April and June 2023. Data were collected using a self-administered questionnaire and analyzed using SPSS version 24. Bivariate and multivariate analyses were conducted using structural equation modeling with AMOS program to examine predictors of dual-protection use.

**Results:**

Of the 1,020 participants, 396 (38.8%) had ever had sexual intercourse, 370 (93.4%) of whom were sexually active in the last 12 months. Of these, only 76 (20.5%) used dual protection at last sex in the past 6 months. Multivariate analysis indicated that dual-protection use was directly and strongly predicted by motivation (*β* = 0.29, *P* < 0.001) and behavioral skills (*β* = 0.27, *P* < 0.001), whereas it was weakly predicted by information (*β* = 0.04, *P* < 0.05), while the information had a strong effect (*β* = 0.27, *p* < .001) on behavioral skills to indirectly influence dual protection use.

**Conclusion:**

These findings support the key roles of motivation and behavioral skills in directly predicting dual protection use, while information can also influence behavioral skills to indirectly affect dual protection use, suggesting the importance of incorporating all elements of the IMB model constructs in designing targeted intervention to promote dual protection behaviors for youth.

## Introduction

Globally, HIV/AIDS and unwanted pregnancies are major public health problems with young women being disproportionately affected. In 2020, of the estimated 1.5 million people newly infected with HIV worldwide, about 260,000 were adolescent girls and young women aged 15–24 years, of these, around 218, 000 (83%) occurred among adolescent girls and young women in sub-Saharan Africa (1–3). In Ethiopia, although the adult HIV prevalence rate declined to 0.93% in 2019, women remained disproportionately affected compared to men (1.22% vs. 0.64%), and the highest prevalence was observed among unmarried young women (9%) (4–7). In addition, like many other countries in sub Saharan Africa, the rate of unwanted pregnancies among young women in Ethiopia remains high, with about 37% of unwanted pregnancies occurring in young women aged 20–24 years in 2011 (6). Recent data from the Ethiopian demographic and health survey (EDHS) also reported that the proportion of young women who begun childbearing before age 20 was approximately 28% in 2016 (8). The fact that adolescent girls and young women in sub-Saharan Africa are substantially affected by the HIV epidemic is because they face several vulnerabilities that increase their risk of unwanted pregnancy and STIs/HIV. These vulnerabilities are rooted in gender roles/social norms, and their limited access to information, education and resources, all of which prevent them from making essential decisions about their health (1–3). As a result, many young women do not receive adequate education about reproductive health, contraception, and safe sex practices, leaving them uninformed about their prevention options and risks. Furthermore, gender inequalies and social norms also place young women in subordinate positions within relationships, making it difficult for them to negotiate safer sex or condom use because they may fear violence from partners if they attempt to assert their preferences regarding condom use (2, 3).

In response to the HIV epidemic, HIV/AIDS prevention programs emphasize safer sexual practices such as abstinence, mutual monogamy, and condom use as effective strategies for reducing the risk of HIV infections among youth (2, 3). In addition, because young women are at increased risk and vulnerable to STI/HIV infections and unwanted pregnancy from the same unsafe sexual practice while both can be prevented simultanousely by the same safer sexual practice as dual protection, the recent WHO/UNAIDS recommendation on HIV prevention for young women emphasize the need for dual protection: defined as safer sexual behaviors that provide simultaneous protection against both HIV/STI and unwanted pregnancy through either abstinence, consistent condom use, or dual-method use (using condom plus hormonal contraceptive), as complementary strategies to maximize prevention efforts for youth in settings with high HIV burden (2, 8). In this study, Dual protection use is defined as simultaneous protection from pregnancy and STI/HIV infection through (either consistent use of a condom alone, or use of two methods (condom plus other contraceptive methods), Consistent condom use is defined as always using a condom in every sexual acts, and Dual-method use is defined as the simultaneous use of a condom for STI/HIV prevention and hormonal methods for pregnancy prevention at the last sex.

In recognition of the problems and vulnerability of unmarried young women to higher risk STI/HIV infections in Ethiopia, the national adolescents’ reproductive health strategy (2007–2015) has stressed the importance of dual protection for unmarried young women to prevent both HIV/STI and unwanted pregnancies simultaneously (6, 7). However, despite the improvements in the rates of contraceptive methods used among unmarried sexually active young women in Ethiopia to 55% in 2016*,* only 4% used condoms (6, 7), suggesting that they are not dually protected and thus remain at an increased risk of HIV infection. Nevertheless, little is known about patterns and predictors of dual protection practices among young women in Ethiopia. In addition, despite the ranges of dual protection options, studies on dual-protection to date almost exclusively has focused on *dual-method use* (using condom for STI/HIV prevention and hormonal contraceptive for pregnancy prevention) measured at a single point in time (e.g., at last intercourse) (9). However, focusing exclusively on dual-method use has important limitations in that it ignores the importance of abstinence and consistent condom use for effective risk reduction approach. Thus, there is a need for understanding patterns and predictors of dual protection use based on theoretical models for designing comprhensive interventions to promote diverse dual protection behaviors among youth at risk of HIVSTI and unintended pregnancy.

### Ethical consideration and informed consent to participate

Ethical approval was obtained from the Institutional Review Board (IRB) of Jimma University with reference No: JUHI/IRB 329/23, date 20/03/2023, which states that “the research protocol meets the ethical and scientific standards outlined in national and international guidelines” in accordance with the Declaration of Helsinki. A formal letter of support was obtained from Mattu University to their respective colleges requesting to cooperate with researchers during the study. Informed consent was obtained from all the participants based on “written consent form” prepared for this purpose and to be signed by all the participants before participating. As all university-level students are older than 18 years, they are believed to be capable of providing informed consent. In addition, the law in Ethiopia does not require such a group of young people to be accompanied by parents or guardians to provide consent to their behaviors, stating that “Assent will be sought from a study participant under the age of 18 years old” (NRERG 7th ed: Art 6.15). Before start, the study's objective was explained to the participants and voluntary participation was allowed by explaining their full rights to participate or not, and to withdraw their consent at any stage if they wish without giving any reason or no penalty. Furthermore, the participants were also assured that the questionnaire was anonymous and that their responses were fully confidential.

### Theoretical perspectives

Various theoretical models have been used to understand HIV risk behaviors and promote safer sexual behaviors across at-risk populations. Of these, the information-motivation-behavioral skills (IMB) model (10–12) is one that has been widely used to understand and promote HIV prevention behaviors. The IMB model asserts that HIV prevention information, motivation, and behavioral skills are fundamental determinants of HIV preventive behaviors (10–12). The model specifies that information and motivation work through behavioral skills to influence HIV-preventive behaviors. The model also assumes that information and motivation may have a direct effect on HIV-preventive behavior when complicated behavioral skills are not required for practice, such as abstinence, as opposed to using condoms by young women (8–10). According to the IMB model, specification of information, motivation, and behavioral skills content most relevant to a specific behavior (abstinence and condom use) and identification of the IMB model constructs that powerfully influence the practice of a specific behavior is crucial for designing empirically targeted interventions to promote a specific sexual health behavior (10, 11, 13, 14).

The IMB model's constructs are also regarded as highly generalizable approach to understanding and promoting sexual health behavior across populations and behaviors of interest (9–11). However, despite the recommendation for its application to a range of sexual health behaviors, there is limited research using the IMB model for understanding predictors of safer sexual dual protection behaviors, such as dual protection use among young women at risk of STI/HIV and unwanted pregnancy.Thus, this study aimed to utilize the IMB model for examining predictors of dual protection use among female university students in Ethiopia for designing targeted interventions to promote dual protection behaviors for this population. The IMB model was chosen because it specifies measurement and statistical procedures for eliciting information, motivation, and behavioral skills factors that are relevant to a particular behaviors (15–18).

### Conceptual framework for the present study

**Figure 1:**
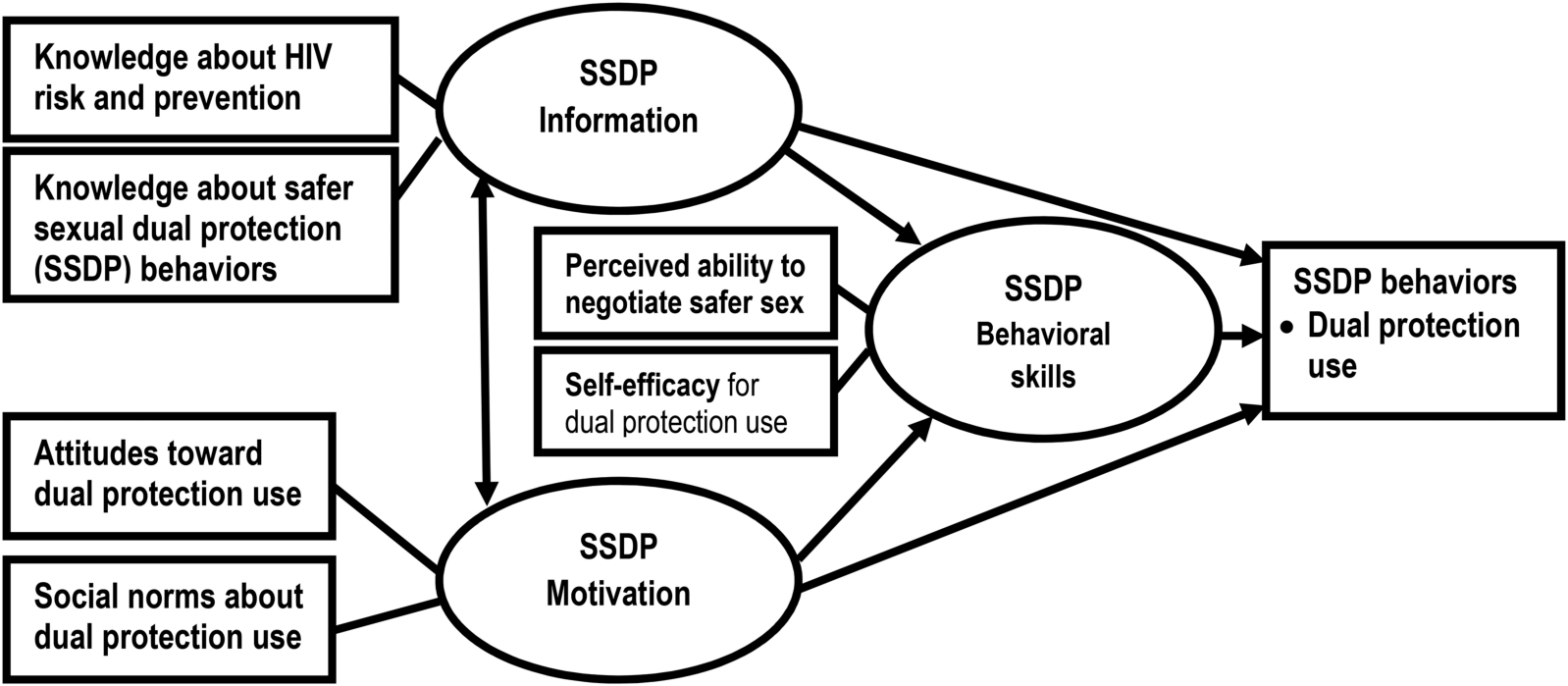
Conceptual framework for the IMB model of the determinants of dual protection use. Source: Adopted from the IMB model of HIV prevention behavior (8–12). Note: Oval represents latent constructs (Information, Motivation and Behavioral skills), Rectangles represent observable variables (Knowledge of HIV risk and safer sexual dual protection behaviors, attitudes and social norms about dual protection use for measuring motivatio and ablity to negociate and self-efficacy). Single-headed arrows represent regression paths, and double-headed arrows represent correlation.

As illustrated in Figure 1, the IMB model for predicting dual protection use posits that an individual's levels of information, motivation, and behavioral skills are determinants of dual protection use. Specifically, possessing specific *information* about the risks of HIV/pregnancy and the benefits of dual protection, *motivation* to engage in dual protection (attitudes toward dual protection use and social norms regarding it) and *behavioral skills* for dual protection (the ability to negotiate with partners and self-efficacy in condom use) will directly predict dual protection use. Furthermore, information and motivation also influence behavioral skills, thereby indirectly affecting dual protection use (see Figure 1).

## Materials and methods

### Study setting, study design and participants

This study was conducted at Mattu University in Ethiopia. Mattu University is a newly established third-generation public university in the country. It has two campuses: the Mattu main campus, located at 551 km southwest of Addis Ababa, and Beddele campus, located at 431 km southwest of Addis Ababa.This study was conducted between April and December 2023.

A cross-sectional survey was used with undergraduate female university students randomly selected from the two campuses of Mattu University. The inclusion criterion includes being unmarried female students and willing to participate and provide informed consent. Whereas married female students, and those who were critically ill were excluded.

### Sample size and its determination

To obtain a sufficient number of participants for numerical analysis, the sample size for the study was calculated for different objectives, and the largest size was taken as the final sample size.

$$n=\frac{2{\left(z_{\alpha /2}+z_{1-\beta }\right)}^{2}}{\frac{{\left(\mu 1-\mu 2\right)}^{2}}{\delta }}=\frac{2{\left(1.96+1.28\right)}^{2}}{{\left(\frac{\Delta }{\delta }\right)}^{2}}=\frac{2(10.51)}{\delta^{2}}=\frac{21}{{\left(0.20\right)}^{2}}=\frac{21}{0.04}=525\times 2=1050$$

Thus, since 1,050 was the largest sample size calculated, it was taken as the final sample size to be used for all objectives, including this study.

### Sampling technique

A multistage sampling technique was used to recruit participants from the two campuses of Mattu University. First, three non-health colleges from each campus were selected using stratified sampling. Then, two departments from each college and two classrooms from each department were selected using cluster sampling in which all female students who fulfilled the inclusion criteria were invited to participate in the study.

### Measurement

To assess the levels of HIV risk reduction/dual protection information, motivation, behavioral skills, and behaviors, a validated measure of the IMB model of HIV prevention behaviors was was adopted from previous research (8, 10, 13, 14, 19, 20).

### A/measures of information

Levels of HIV risk reduction and dual protection information were measured with 12 items, using T/F responses. a) Knowledge about HIV risk and prevention was assessed with 8 items, such as (1) “HIV can be transmitted through sexual intercourse”; 2) “Abstinence is the most effective way of avoiding risk of STI/HIV”; or 3) “Consistent condom use is an effective method of preventing STI/HIV,”. b) Knowledge about safer sexual dual protection behaviors was assessed with four items, such as (1) “Abstinence is the best way to avoid the risk of HIV/STI and pregnancy for unmarried youth”; (2) “Consistent condom use prevents HIV/STI and pregnancy, providing dual protection against both”; and (3) “Dual methods use is options for dual protection against both HIV/STI and pregnancy simultaneously” (8, 10, 13, 14, 19, 20).

### B/measures of motivation

Motivation to practice dual protection use was assessed based on attitudes and social norms regarding dual protection, using 5 pionts Likert scale: *a) Attitudes toward condom use* were assessed with 4 items: (e.g., 1) “condoms do not interfere with sexual enjoyment,” 2) “consistent condom use is effective for preventing HIV/STI and pregnancy”, and 3) “condoms should always be used, even if a woman is using hormonal contraception.” b) Social norms about condom use were assessed with two items: (1) “The belief that condom use may show my partner that I do not love/trust him” and 2) “If I ask my partner to use condoms, he would suspect me that I have another partner and that he would become angry at me” (8, 10, 13, 14, 19, 20).

### C/measures of behavioral skills

Behavioral skills for dual protection use were assessed based on perceived ability and Self-efficacy for dual protection, using 5 pionts Likert scale: *a) Perceived ability to negotiate with one's partner* was assessed with two items: (e.g., 1) “How confident are you in your ability to negotiate with partners to use condoms consistently?” and 2) “How confident are you in using condoms consistently even if you are using hormonal contraception?”). *b) Self-efficacy in using condoms* was assessed with two items: (e.g., 1) “If your steady partner doesn't want to use a condom, how certain are you that you could convince him to use condoms?”; 2) “If you are already using another method of contraception for pregnancy, how certain are you that you could always use condoms for reducing the risk of HIV?” (8, 10, 13, 14, 19, 20).

### D/measures of safer sexual dual protection behaviors

Sexual dual-protection behaviors were assessed using self-reported protection method used.

1) Primary sexual abstinence was assessed by asking “Have you ever had sex to date?” with a response (yes or no).
2) Dual protection use was assessed by asking, “Did you use the dual protection method in your last sex in the past 12 months”?, with a response option (yes/no).
3) Consistent condom use was assessed by asking “How frequently did you use condoms in the past 12 months?” with responses options (1) “always” to (2) “never.”
4) Dual method use was assessed by asking “Did you use condoms plus another contraceptive method in the past 12 months” with responses (yes or no).

### Data collection procedure

Data collection was carried out in their classrooms, where students were asked to fill out an anonymous questionnaire prepared in English and then translated into Afaan-Oromoo and Amharic languages, for better understanding of the concepts of each question. The questionnaires were administered by 4 research teams who had a B.Sc degree, but not staff of the same university, and they were trained as supervisors to help students complete the questionnaire, to explain the purpose of the study, clarify questions whenever necessary, and collect the completed questionnaires at the end. A pre-test of the questionnaire was administered to 5% of the sample, with a total of 51 students, but not taking part in the study.

### Data analysis

The collected data was cleaned, edited, and analyzed using SPSS version 23. Descriptive statistics were used to summarize the distribution of participants based on their demographic characteristics and the level of HIV risk reduction/dual protection information, motivation, behavioral skills, and behaviors. Bivariate and multivariate analysiswere performed using structural equation modeling (SEM) with AMOS 24 (17, 18) to examine correlates and predictors of dual-protection use. Spearman's correlations were used to examine correlations among the indicators of the IMB model and dual protection use. Estimating the parameters of the measurement model (confirmatory factor analysis) and the structural model (path analysis) was accomplished with a maximum likelihood estimate in AMOS. Confirmatory factor analysis (CFA) was used to examine the relationships between the observed variables and latent constructs in the model (i.e., to test the reliability and validity of the measurement model). Path analysis was used to examine the relationships between the latent constructs in the structural model (i.e., the information, motivation and behavioral skills constructs) and identify the predictors of dual protection use.

## Results

### Descriptive statistics

#### i/ Sociodemographic characteristics and sexual behaviors of the study participants

As shown in Table 1, of the 1,020 participants, 820 (80.4%) were aged 20–24 years, with a mean age of 20.77 years (SD = 1.146). Regarding ethnicity, 526 (51.6%) were Oromos, followed by Amhara (356; 34.9%). According to religion, 442 (43.3%) were Orthodox, 364 (35.7%) were Protestants, 180 (17.6%) were Muslims, and 34(3.3%) were Waaqeffanna followers. With respect to sexual behaviors, 624 (61.2%) reported never having sexual intercourse, whereas 396 (38.8%) had ever had sexual intercourse. Among sexually active in the past 12 months (*n* = 370), about 175 (47.3%) used condoms at last sexual encounter, while only 20.5% used dual protection methods in the past year, with 22 (5.9%) using condoms alone, and 54 (14.6%) using two-methods (condom plus hormonal methods) at their last sex (see Table 1).

**Table 1:** Distribution of the participants by demographic characteristics and sexual behaviors (*n* = 1,020).

Demographic characterstics		*n*	%
Age group	15–19	170	16.7
	20–24	820	80.4
	25–29	30	2.9
Ethnic groups	Oromoo	526	51.6
	Amhara	356	34.9
	Sidama	111	10.9
	Others^a^	27	2.6
Religious group	Waaqeffachuu	34	3.3
	Protistant	364	35.7
	Ortodox	442	43.3
	Musilims	180	17.6
Place of residence	Urban	466	45.7
	Rural	554	54.3
Sexual practice and dual protection use			
Ever had sexual intercourse (*n* = 1,020)	Yes	396	38.8
	No	624	61.2
Sexual activity in the past 12 months (*n* = 396)	Yes	370	93.4
	No	26	6.6
Condom use at last sex in the past 12 months (*n* = 370)	Yes	175	47.3
	No	195	52.7
Use of dual protection for preventing STI/HIV and pregnancy in the past 12 months (*n* = 370)	Yes	76	20.5
	No	294	79.5
Types of dual protection methods used (consistent condom se alone or dual methods use) (*n* = 76)	Consistent condom use	22	5.9
	Two methods use	54	146

^a^ Represents those of other ethnic groups including Gorage, Walayitta, Tigre and Hadiya.

#### ii/ Levels of HIV risk reduction/dual protection information, motivation and behavioral skills among sexually active female university students (*n* = 396)

As presented in Table 2, among sexually active participants (*n* = 396), 207 (52.3%) had high knowledge of HIV risk and prevention and 220 (55.6%) had high knowledge of safer sexual dual protection behaviors. Regarding motivation to practice dual protection use among sexually active participants, only fewer than half, 173 (43.7%) had positive attitude toward dual protection use and 157 (39.6%) had supportive norms for dual protection use. With regrad to behavioral skills required for dual protection use, nearly half, 195 (49.2%) had high self-efficacy in negotiating safer sex with partners (see Table 2).

**Table 2:** Descriptive statistics of the levels of HIV risk reduction/dual protection information, motivation, behavioral skills among sexually active female students (*n* = 396).

IMB constructs	Levels	*n*	%	Mean	SD	*α*
Information						
Knowledge about HIV risk and prevention	High	207	52.3	6.18	1.395	.56.
	Low	189	47.7			
Knowledge about safer sexual dual protection behaviors	High	220	55.6	2.79	1.023	.66
	Low	176	44.4			
Motivation						
Attitudes toward dual protection use	Positive	173	43.7	3.3	1.84	.97
	Negative	223	56.3			
Social norms about dual protection use	Supportive	157	39.6	3.44	1.48	.89
	Unsupportive	239	60.4			
Behavioral skills						
Percieved ability/self-efficacy in dual protection	High	195	49.2	3.57	1.58	.91
	Low	201	50.8			

### Bivariate correlations analysis among indicators of IMB model and dual protection use

As shown in Table 3, results of Spearman's correlations analysis showed that the informational indicators of knowledge about HIV risk reduction and safer sexual dual protection behaviors were strongly correlated(r = .722, *p* < .001). In addition, the informational indicators are also correlated with the motivational indicators of attitudes towards dual protection(r = .597, *p* < .001) and social norms about dual protection use (r = .547, *p* < .001), and also with the behavioral skill indicators of self-efficacy in using dual protection (r = .508, *p* < .001).Similarly, the motivational indicators of *attitudes* toward dual protection use and *social norms* about dual protection were strongly correlated (r = .910, *p* < .001), and both are also correlated with the behavioral skill indicators of self-efficacy for dual protection use (r = .637, *p* < .001) and (r = .565, *p* < .001) respectively.

**Table 3:** Correlational analysis among indicators of IMB model constructs and dual protection use among sexually active female university students (*n* = 396).

Spearman's Correlations							
IBM indicator variables		1	2	3	4	5	6
1	Knowledge about HIV risk reduction	1	.	.	.		
2	Knowledge about ssdp behaviors	.722^a^	1	.			
3	Attitude towards dual protection use	.597^a^	.542^a^	1	.	.	
4	Social norms about dual protection use	.547^a^	.476^a^	.910^a^	1	.	
5	Percieved self-efficacy for dual protection	.508^a^	.471^a^	.637^a^	.565^a^	1	
6	Dual protection use	.332^a^	.337^a^	.477^a^	.372^a^	.501^a^	1

^a^Correlation is significant at the 0.01 level (2-tailed).

Overall, the bivariate analysis showed that dual protection use was strongly correlated with knowledge about HIV risk reduction (r = .332, *p* < 0.01), knowledge of safer sexual dual protection behaviors (r = .337, *p* < 0.01), attitude towards dual protection use (r = .477, *p* < 0.01), social norms about dual protection use (r = .372, *p* < 0.01) and perceived self-efficacy for using dual protection (r = .501, *p* < 0.001) (see Table 3).

### Multivariate analysis

The extent to which the data conformed to the theoretical model was assessed using the model's fit indices that revealed the Chi-squared test value (X2 = 11.399, *P* = .220), comparative fit index (CFI = .99), and root mean square error of approximation (RMSEA = .048), all indicating the model fit was acceptable.

### Confirmatory factor analysis (CFA)

As presented in Table 4, the maximum likelihood estimates of the measurement model showed that all the factor loadings for the relationships between the observed variables and latent constructs were strong and statistically significant, suggesting reliability of the model. Specifically, the standardized factor loadings (*β* = 0.89) for knowledge of HIV risk and prevention and (*β* = 0.82) for knowledge of safer sexual dual protection behaviors on the information construct suggest their strong and significant relationships. In addition, the standardized factor loadings (*β* = 1.1) for *attitudes* toward dual protection use and (*β* = 0.89) for *social norms* about dual protection on the motivation construct also suggest strong and significant relationships. Finally, the standardized factor loadings (*β* = 1.1) for perceived ability to negotiate safer sex and (*β* = 0.86) for self-efficacy in using dual protection on the behavioral skill construct indicated strong and significant reliability (*P* < 0.001) (see Table 4).

**Table 4:** Maximum likelihood estimates of the measurement model parameters for the IMB model of dual protection use among sexually active female university students (*n* = 396).

Indicator/variables			Estimate	S.E.	t.	*P*	Standardized factor loading (β)
Information	–>	Knowledge of HIV risk & prevention	1.094	.070	15.681	***	.89
	–>	Knowledge of safer sexual dual protection behavior	1.000				.82
Motivation	–>	Attitude toward dual protection use	1.155	.038	30.643	***	1.1
	–>	Social norms about dual protection use	1.000				.89
Behavioral skills	–>	Percieved ability to negotiate safer sex	1.000				1.1
	–>	Self-efficacy for using dual protection	.943	.039	24.154	***	.68

Note: Indicators with factor loadings of 1.000 set the metric of the construct.

*** *p* < 001.

### Path analysis

**Figure 2:**
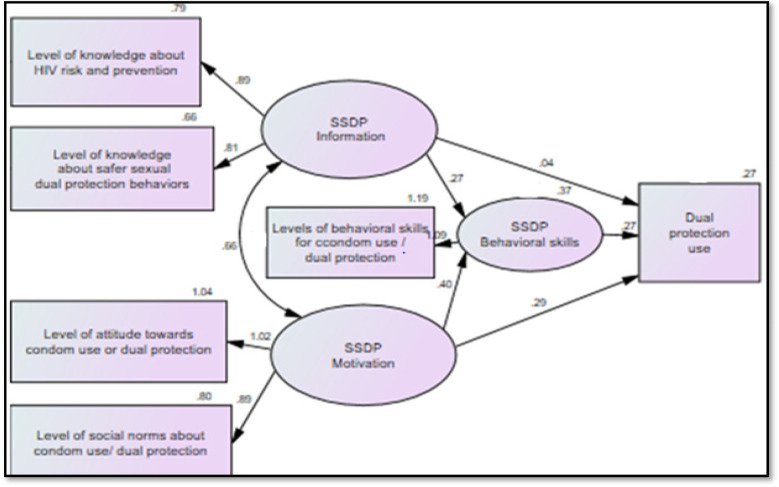
Significant regression paths in the IMB model predictors of dual protection use among sexually active female university students (*n* = 396). Source: Adopted from the IMB model of HIV prevention behavior (8–12). Note: Oval represents latent constructs, and rectangles represent observable variables. Single-headed arrows represent regression paths, and double-headed arrows represent correlations. Regression coefficients and covariance estimates are standardized.

As shown in Table 5 and Figure 2, the results of path analysis indicated that information (*β* = 0.27, *P* < .001) and motivation (*β* = 0.40, *P* < .001) were significantly associated with behavioral skills, and that behavioral skills, in turn, were reliably associated with dual protection use (*β* = 0.27, *P* < 0.001). In addition, the direct effect of motivation on dual protection use was strong (*β* = 0.29, *P* < 0.001), whereas the direct effect of information on dual protection use was moderate (*β* = 0.04, *P* < 0.05). Overall, the full model's constructs accounted for approximately 27.4% of the variation in dual-protection use, while the information and motivation constructs explained about 37% of the variation in behavioral skills (see Table 5 and Figure 2).

**Table 5:** Estimates of the structural parameters for the IMB model of dual protection use amongnsexually active female university students (*n* = 396).

Parameters			Estimate	S.E.	t.	*P*	Standardized path coefficient	R2
Information	–>	Behavioral skills	.356	.074	4.820	***	.27	.37
Motivation	–>	Behavioral skills	.495	.062	7.945	***	.40	
Information	–>	Dual protection use (DPU)	.039	.060	1.351	.025	.04	.27
Motivation	–>	Dual protection use (DPU)	.270	.222	2.443	***	.29	
Behavioral skills	–>	Dual protection use (DPU)	.199	.044	4.414	***	.27	
Information	<–>	Motivation	.116	.013	9.001	***	.656	

Note: R2 = represents the proportion of explained variance in the dual protection use that is accounted for by the IMB model constructs.

***p < 0.001.

## Discussion

The current study utilized the IMB model for understanding predictors of dual protection use among female university students in Ethiopia. The results showed that significant proportion of female university students were engaged in risky sexual behaviors, such as premarital sex with low use of condom/dual protection that may place them at increase risks for STI/HIV infections. In this study, only 20.5% of sexually active female students have used dual protection methods during intercourse in the past 12 months, with 22 (5.9%) using condoms alone, and 54 (14.6%) using two-methods (condom plus hormonal methods) at their last sex. This finding is in line with those of studies conducted in the United States, where use of dual protection among sexually active adolescents ranged from 14% to 25% (21). Nevertheless, the results of our study are lower than those of studies in Canada, where 30% of sexually active Grade 11 students reported using dual protection at last sex (8). The differences in culture and values might be the cause of these results’ disparity.

In addition, though over two-third of the participants reported to have higher knowledge about HIV risk and prevention, the majority of participants still have a significant knowledge gaps about safer sexual dual protection behaviors, have a negative attitude towards dual protection use, and lower self-efficacy for using dual protection in this study. The findings of this study is consistent with the results of studies conducted among WolaitaSodo University students and Mattu secondary school students in Ethiopia, where many students had unfavorable attitude towards safer sex and inadequate skills for practicing abstinence (17, 22). However, research suggests that knowledge of HIV risk and safer sexual behaviors is crucial to enable people to avoid HIV infection, especially for youth, who are often at greater risk because they may have shorter relationships and thus more partners or may engage in other risky behaviours. In addition, levels of attitudes and skills for practicing safer sex can affect one's practice of sexual behaviors.

Regarding the determinants of dual protection use among sexually active young women, the results of bivariate correlational analysis between indicators of IMB model construct and dual protection use in this study showed that dual protection use is strongly associated with knowledge of safer sexual dual protection behaviors, attitude towards dual protection use, perceived social support for dual protection, and self efficacy to negotiate safer sex with partners. This is consistent with the results of previous studies on the determinants of dual protection use among young women in Canada, which indicated that several factors including social, cultural and psychological factors were correlated with dual methods use (9).

The results from the SEM analysis of IMB model in predicting dual protection use also indicate that information and motivation were independent factors, and each was positively and significantly associated with behavioral skills, and that behavioral skill, in turn, were reliably associated with dual protection use. This finding is consistent with those of previous studies conducted among urban adolescents in the United States (19), among college and university students in the United States (20, 23) and among urban high school youth in the United States (15), among young adults in high HIV burden districts, south Africa (24), among Aferican-American college students in the United States (25), among women in low income housing (26), and underserved minority youth in the United States (27–31) that tested the IMB model's hypothesis that information and motivation are significantly associated with behavioral skills and that behavioral skills, in turn, are associated with HIV-preventive behavior (condom use) (19–21, 23–27). Together, the model constructs accounted for approximately 27.4% of the variance in dual-protection use. In addition, the findings also indicate that information and motivation have reliable effects on behavioral skills, with both constructs explaining about 37% of the variation in behavioral skills. These findings are congruent with the findings of previous studies, in which the model's constructs accounted for one-third to one-half of the variance in condom use) (19–21, 23–27). The results also support the IMB model as a paradigm for explaining and predicting sexual health dual protection of young women.

### Limitations

This study has several limitations. First, the study focused only on female university students for the purpose of examining patterns and predictors of dual protection use to simultaneously prevent both STI/HIV and unwanted pregnancy, excluding male students for priority concern. Second, the data based on self-reported sexual behaviors may lead to bias arising from socially desirable responses, because sex remains a sensitive topic in Ethiopian culture. However, the use of self adminstared data collection methods and anounmous questioniiares is intended to reduce such bias.Third, the data from the cross-sectional survey can reveal only the associations between variables at a single point in time; thus, it is recommended that future research should focus on longitudinal studies to test the model.

## Conclusions

The current research represents the first attempt to utilize the IMB model for understanding psychological determinants of safer sexual dual protection behaviors among youth in Ethiopia. The findings indicated that a sizable percentage of female university students are engaged in risky sexual practices with low use of dual protection, while the majority of participants lack adequate knowledge of safer sexual dual protection behaviors, favorable attitudes toward dual protection use, and self-efficacy for using dual protection. The results of correlations analysis indicated that predictors of dual protection use were knowledge of safer sexual dual protection behaviors, attitude towards dual protection use and self-efficacy for using dual protection.

These results have significant practical implications, especially for young women at risk of STI/HIV infections even when using hormonal contraception to prevent pregnancy without use of condoms/dual protection in settings with high HIVburdens. By focusing on the application of the IMB model to predict dual protection behavior, this study provides novel insights into the interplay between information, motivation, and behavioral skills in promoting safer sexual dual protection behaviors among youths. These insights will not only advance our theoretical understanding of sexual behavior of youth in high HIV burden settings, but also inform the development of comprhensive interventions that address the diverse needs of young people. Finally, it is recommended that future IMB model test should focus on a search for possible sex differences in the determinants of safer sexual dual protection behaviors between males and females.

## Data Availability

The original contributions presented in the study are included in the article/Supplementary Material, further inquiries can be directed to the corresponding author.
